# Enhanced catalytic activity without the use of an external light source using microwave-synthesized CuO nanopetals

**DOI:** 10.3762/bjnano.8.118

**Published:** 2017-05-30

**Authors:** Govinda Lakhotiya, Sonal Bajaj, Arpan Kumar Nayak, Debabrata Pradhan, Pradip Tekade, Abhimanyu Rana

**Affiliations:** 1 Material Science Center, Indian Institute of Technology Kharagpur, Kharagpur-721302, W.B., India; 2 Jankidevi Bajaj College of Science, Wardha-442001, M.S., India; 3 MESA+ Institute for Nanotechnology, University of Twente, 7500 AE, Enschede, Netherlands

**Keywords:** CuO nanopetals, dark catalytic activity, fast degradation of dyes, microwave synthesis

## Abstract

We report enhanced catalytic activity of CuO nanopetals synthesized by microwave-assisted wet chemical synthesis. The catalytic reaction of CuO nanopetals and H_2_O_2_ was studied with the application of external light source and also under dark conditions for the degradation of the hazardous dye methylene blue. The CuO nanopetals showed significant catalytic activity for the fast degradation of methylene blue and rhodamine B (RhB) under dark conditions, without the application of an external light source. This increased catalytic activity was attributed to the co-operative role of H_2_O_2_ and the large specific surface area (≈40 m^2^·g^−1^) of the nanopetals. We propose a detail mechanism for this fast degradation. A separate study of the effect of different H_2_O_2_ concentrations for the degradation of methylene blue under dark conditions is also illustrated.

## Introduction

Controlling air quality and water pollutants is a big challenge for environmental research [1]. Particularly, efforts have been taken to control these pollutants with the development of cost effective and ecologically friendly methods [2]. Metal oxides have attracted significant attention as a photocatalyst for the degradation of these pollutants [3–6]. Copper oxide (CuO) is one of the most efficient materials for the oxidation of the air pollutant carbon monoxide (CO) [7–9]. CuO is one of the few p-type metal oxide semiconductors with a narrow band gap ≈1.24 eV [10]. The properties of CuO nanomaterials (nanoparticles, nanowires, nanosheets, etc.) are closely related to morphology and crystallite size [7]. These different nanoscale morphologies enhance the photoconductive and photochemical properties in various energy applications [7]. Being inexpensive, nontoxic, and readily available, CuO has attracted particular attention. However, in the degradation of water pollutants (e.g., industrial dyes) as a photocatalytic oxidative species, CuO is found to be less effective as compared to other metal oxides [8–12]. Thus, in order to enhance its photocatalytic activity, CuO can be used with hydrogen peroxide (H_2_O_2_) [12–21]. However, the degradation time of dyes is an important problem when using CuO as the photocatalyst.

Here, we have adopted the simple microwave-assisted route for the wet chemical surfactantless synthesis of copper oxide (CuO) nanostructures (nanoflowers and nanopetals) having a large specific surface area. The catalytic reaction of CuO nanopetals and H_2_O_2_ was studied under the application of an external light source and also under dark conditions for the degradation of hazardous dyes such as methylene blue and rhodamine B. We report enhanced catalytic activity of the synthesized CuO nanopetals, even without the use of an external light source (UV/visible light) for the degradation of these dyes. This is attributed to the role of H_2_O_2_ and the large specific surface area of the nanopetals. The amount of the catalyst (CuO nanopetals) and the hazardous H_2_O_2_ is minimized, and the reproducibility of the degradation of the dye with the same catalyst has been tested. The catalytic activity of CuO nanopetals activated by irradiation with photons (visible light) in the absence of H_2_O_2_ is also studied and compared with the activity under dark conditions.

## Results and Discussion

### Structural and morphological study

**Figure 1:**
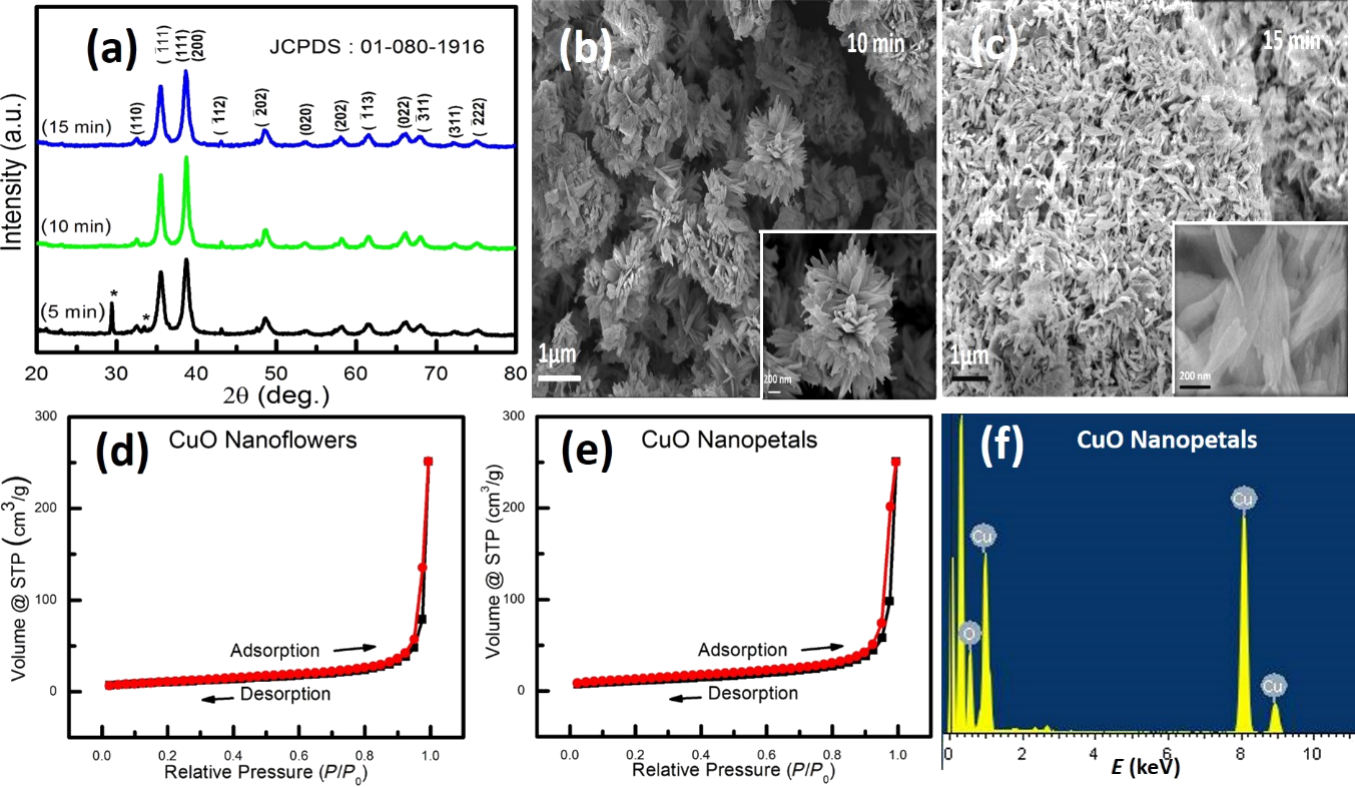
(a) XRD patterns of CuO nanomaterials synthesized by varying the reaction duration of 5 min, 10 min, and 15 min. FESEM images of CuO nanostructures obtained for the synthesis duration of (b) 10 min and (c) 15 min. The scale bar for the insets in (b, c) is 200 nm. Nitrogen adsorption–desorption isotherm for (d) CuO nanoflowers and (e) CuO nanopetals measured at 77 K. (f) EDAX spectra of CuO nanopetals.

Figure 1a illustrates X-ray diffraction (XRD) patterns of CuO nanomaterials synthesized by varying the reaction duration of 5, 10, and 15 min. The sample obtained after 5 min of reaction time shows the diffraction peaks of both CuO and Cu_2_O (marked by *), indicating mixed-phase growth. It is interesting to observe that with the increase in the reaction duration, the diffraction peaks for Cu_2_O disappear. For the samples obtained after 10 and 15 min of reaction time, the XRD pattern matches with JCPDS card no. 01-080-1916, which confirms the formation of phase-pure monoclinic CuO. The average crystallite size for the samples obtained after 10 and 15 min was calculated by using the Scherrer equation and is estimated to be ≈11 nm. As the sample obtained after 5 min exhibits phase impurity, only samples obtained with the reaction duration of 10 and 15 min were considered for further characterization.

Figure 1b,c shows field-emission scanning electron microscope (FESEM) images of CuO nanostructures synthesized with microwave irradiation for 10 min and 15 min. The CuO sample obtained with the reaction time of 10 min was found to resemble a flower-like morphology. Increasing the reaction duration to 15 min resulted in distinct and individual, uniform features having a petal-like morphology, which is clearly visible in Figure 1c. The average width and length of these petal-like features are measured to be 250 and 400 nm, respectively. The insets of Figure 1b,c show the corresponding, magnified image, illustrating their size and morphology. The prolonged microwave agitation is believed to provide the necessary thermal energy for the morphological transformation. This can be inferred on the basis of the similarity in the morphological parameters of both the nanostructures. The growth of these surfactant-free nanostructures depends on certain parameters, including the concentration of NaOH, which promotes the preferential growth of CuO primary crystals along the <010> and suppresses the growth in <001> [22–23]. This preferential growth of the CuO nanostructure has also been observed in the sample obtained after a reaction time of 5 min, where some flake-like morphology is formed (Supporting Information File 1, Figure S1).

### Surface study

The Brunauer–Emmett–Teller (BET) technique was used to measure the surface area, pore radius, and pore volume of the CuO nanoflowers and nanopetals obtained by microwave synthesis at a duration of 10 and 15 min, respectively. Figure 1d,e shows nitrogen adsorption–desorption plots for the CuO nanoflowers and nanopetals. The effective specific surface area, pore radius and pore volume of nanoflowers (and nanopetals) were measured to be 37.2 m^2^·g^−1^ (39.87 m^2^·g^−1^), 17.48 Å (17.646 Å), and 0.38 mL·g^−1^ (0.38 mL·g^−1^), respectively. The marginal increase in the surface area of nanopetals as compared to nanoflowers supports the hypothesis of disintegration of nanoflowers into nanopetals with increasing reaction duration. It should be noted that the specific surface area of the products in the present work is larger than that of previous reports on materials with similar morphology [24]. Figure 1f depicts the energy disperse X-ray photon spectroscopy (EDS) spectra of nanopetals of CuO, which confirms the stoichiometry and atomic percent of the synthesized material. The effective specific surface area of the sample obtained after a reaction duration of ≈5 min was found to be ≈25.58 m^2^·g^−1^ (Supporting Information File 1, Figure S2), which is in good agreement with the preferential growth of nanocrystals along a certain direction with respect to time. As the effective surface area of as-synthesized nanopetals was larger than that of as-synthesized nanoflowers with the same morphology, further experiments were performed with as-synthesized nanopetals.

### Optical study

**Figure 2:**
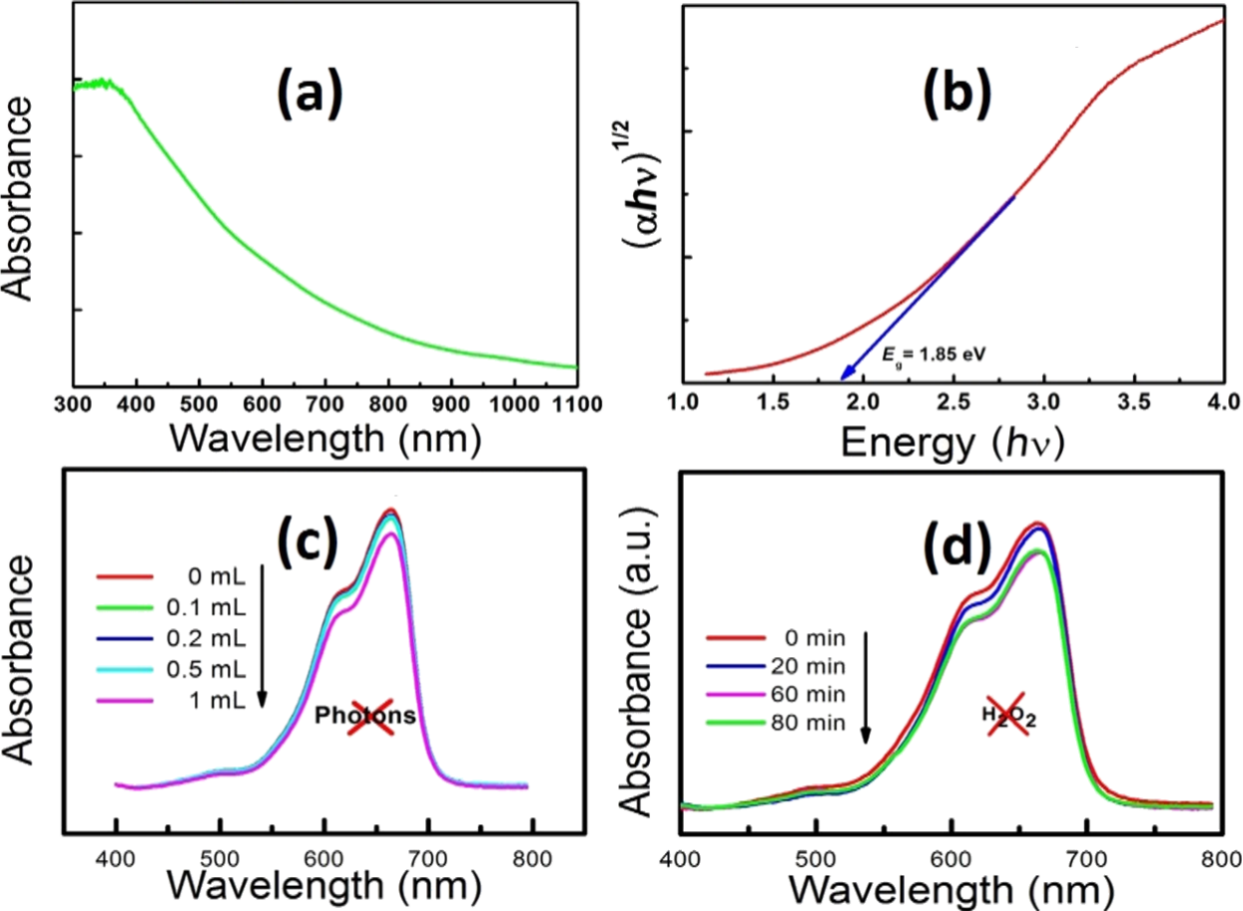
(a) UV–vis absorption spectra of CuO nanopetals. (b) Tauc’s plot for CuO nanopetals (c) UV–vis absorption spectra illustrating the effect of different concentrations of H_2_O_2_ in methylene blue degradation in the absence of CuO nanopetals (catalyst) under dark conditions. (d) UV–vis absorption spectrum of methylene blue degradation using CuO nanopetals as a photocatalyst for different durations.

The UV–vis absorption spectra of as-synthesized CuO nanopetals is shown in the Figure 2a. It reflects a wide absorption spectrum up to 700 nm covering almost the entire visible spectrum. The absorption onset was estimated from the Tauc’s plot as shown in Figure 2b. The band gap of CuO nanopetals was calculated by extrapolating the linear part of the plot of (α*h*ν)^1/2^ vs *h*ν and is found to be ≈1.85 eV as shown in Figure 2b. This is different from the bulk bandgap of CuO, which is 1.24 eV [10]. This blue shift in the absorption further confirms the nanometer range of synthesized material.

### Catalytic activity of nanopetals for the degradation of methylene blue

The use of inorganic semiconductors as a heterogeneous, photocatalytic material has been extensively investigated under UV–vis light illumination, yet continues to attract even more attention due to the use of advanced materials in the process [3–5]. The catalytic photo-degradation of dyes takes place with the excitation of a catalyst using UV–vis light, leading to the generation of electrons and holes which are further responsible for the degradation through the formation of radicals [6,25]. The wide band gap, high surface area of CuO nanopetals was expected to be suitable for the photocatalytic activity for the degradation of the common cationic dye methylene blue (MB), and hence initially, a study has been carried out in which 40 mg of CuO nanopetal powder was dispersed in 40 mL of 50 µM MB solution. This solution was allowed to stir for ≈30 min under dark conditions and was then subjected to irradiation using an incandescent lamp (Philips, 200 W) at a working distance of 100 cm. Aliquots of about 4 mL were taken from the suspension at regular intervals and were centrifuged to filter suspended CuO powder. The MB concentration in the filtered suspension was studied with a Perkin Elmer Lambda 750 UV–vis spectrophotometer. Figure 2b shows the UV–vis absorption spectra of the aliquots taken out at different time intervals. In this study, the MB degradation rate is found to be very slow and only ≈10% degradation has been observed in three hours. No further noticeable bleaching was observed within the next hour. This slow rate of degradation is in agreement with Miyauchi et al. [13], which may be due to the more negative valence band position of CuO than that of the redox potential required for producing free radicals for effective degradation. This slow rate of degradation was then overcome by introducing H_2_O_2_ along with CuO, which resulted in the enhancement of the degradation of pollutants [12,26]. Recently, Zhang et al. successfully enhanced the catalytic activity by using peroxymonosulfate in their system instead of H_2_O_2_ [6]. Few reports are available in which degradation of water pollutants were studied with CuO and H_2_O_2_ without photon irradiation (UV/visible) [27–28]. Therefore, a separate study was carried out to investigate the effect of different concentrations of H_2_O_2_ for MB degradation in the absence of CuO nanopetals (catalyst) without any irradiation (UV/visible light). Figure 2c shows the UV–vis absorption spectra for different concentrations of H_2_O_2_ in MB solution, without catalyst, after one hour without photon irradiation. As can be seen from the Figure 2c, the concentration of H_2_O_2_ with less than 1 mL had almost no effect on the degradation of the dye. However, the higher concentration of H_2_O_2_ alone was found to be effective to some extent in the degradation of dye (6% in 1 h) even in the absence of catalyst. Furthermore, an experiment was performed by adding 1 mL of H_2_O_2_ to a solution containing 40 mg CuO nanopetals in 40 mL of 50 µM MB solution. The solution went from a bluish color (MB solution) to colorless within two minutes. This fast activity without any irradiation unveils the interesting co-operative role of CuO and H_2_O_2_ for the degradation of MB.

**Figure 3:**
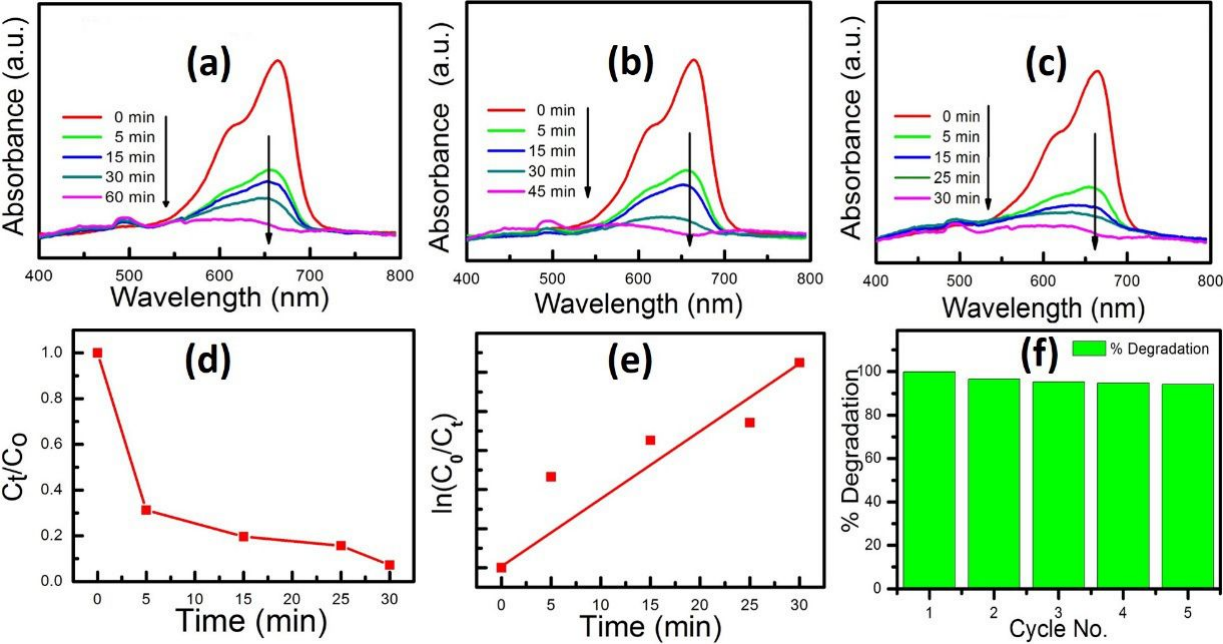
(a) UV–vis absorption spectra for MB degradation for different duration for 10 mg nanopetals of CuO and 0.1 mL H_2_O_2_. (b) UV–vis absorption spectra for MB degradation for different duration for 10 mg nanopetals of CuO and 0.2 mL H_2_O_2_. (c) UV–vis absorption spectra for MB degradation for different duration for 10 mg nanopetals of CuO and 0.5 mL H_2_O_2_. (d) The MB degradation rate in the presence of 0.5 mL H_2_O_2_ and 10 mg CuO nanopetals. (e) Kinetics of degradation with 0.5 mL H_2_O_2_ and 10 mg CuO nanopetals. (f) The % degradation vs cycle number for MB degradation with 0.5 mL H_2_O_2_ and 10 mg CuO nanopetals.

Further experiments were focused to achieve a higher rate of MB degradation with the optimum use of the cost effective catalyst and the hazardous H_2_O_2_. The amount of CuO nanopetals was minimized and fixed at 10 mg and concentrations of H_2_O_2_ were varied (0.1 mL, 0.2 mL and 0.5 mL) to study the catalytic activity for the degradation of 40 mL of 50 µM MB solution without irradiation. Figure 3a–c depicts the UV–vis absorption spectra of MB aliquot using 0.1 mL, 0.2 mL and 0.5 mL of H_2_O_2_, respectively, with 10 mg of catalyst. It can be clearly observed that the concentration of H_2_O_2_ has an obvious effect on the degradation time, which decreases with increasing H_2_O_2_ concentration.

As can be seen from Figure 3c, the complete degradation of the dye within 30 min was successfully achieved with 0.5 mL H_2_O_2_ along with 10 mg CuO nanopetals. Figure 3d represents the kinetics of the MB degradation with 0.5 mL H_2_O_2_ and 10 mg CuO nanopetals. The apparent rate constant of this degradation was calculated from the slope of ln(*C*/*C*_0_) vs time (Figure 3e) and found to be 0.087 min^−1^. In order to examine the stability of the catalyst used for MB degradation, experiments with 0.5 mL H_2_O_2_ and 10 mg nanopetals were repeated five times using the same CuO nanopetals without irradiation. Figure 3f shows the efficiency of the catalyst (five continuous cycles) in which degradation was efficiently achieved within 30 min. In the fifth cycle, the same catalyst could still successfully degrade ≈94% of MB dye in 30 min. This highlights the efficacy and high stability of CuO nanopetals as a catalyst in this dye degradation activity. The phase and morphology of CuO nanopetals after the fifth cycle was also confirmed by XRD and FESEM (not shown).

The catalytic activity of the sample obtained after 10 min (nanoflowers), whose surface area was closer to nanopetals, was also checked in the presence of H_2_O_2_ under dark conditions. For this, 10 mg of CuO nanoflowers along with 0.5 mL of H_2_O_2_ was used in a 40 mL, 50 µM solution of MB. As expected, the nanoflowers could efficiently degrade ≈93% of MB in 30 min (Supporting Information File 1, Figure S3).

### Reaction mechanism

It is well understood that the rate of degradation of organic dyes depends on the formation of free radicals [7,25,28]. The fast degradation of MB with the assistance of H_2_O_2_ and as-synthesized CuO nanostructures (petals/flowers) without irradiation with photons is believed to be the co-operative phenomenon and proceeds through the following two vital steps:

[1]
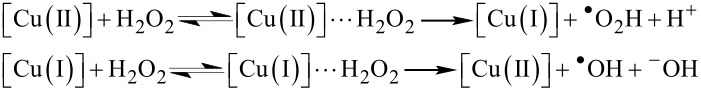


[2]
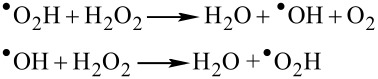


At first, H_2_O_2_ reacts with the complex surface of nanopetals [Cu(II)] and reduces it to produce free radical ^•^O_2_H and species [Cu(I)], which again, upon reaction with H_2_O_2_, become oxidized to give back [Cu(II)] along with radical ^•^OH, as represented in Equation 1. Thus, the free radicals ^•^OH and ^•^O_2_H are generated in the reaction solution due to the oxidizing and reducing property of H_2_O_2_. These free radicals may be adsorbed on H_2_O_2_ and can produce each other as depicted in Equation 2. Then, these free radicals ^•^OH and ^•^O_2_H, which have a very high oxidizing capability, interact with the S atom in the middle heterogeneous ring of MB dye. This leads to the very easy degradation of the dye and produces SO_4_^2−^ ions (the main product of MB oxidation) [29]. The formation of SO_4_^2−^ ions in the mineralized degraded product was confirmed by adding BaCl_2_ to it, which produces the white color precipitate of BaSO_4._ Here, the larger surface area of CuO nanopetals provides higher adsorption of H_2_O_2_ molecules for more radical formation to enhance the rate of degradation. Thus, the complete degradation reaction of MB under dark conditions was achieved in ample time and found to proceed through the co-operative activity between CuO nanostructures and H_2_O_2_.

### Catalytic activity of nanopetals for the degradation of rhodamine B

The catalytic performance of synthesized CuO nanopetals was also extended for the degradation of rhodamine B (RhB) using 40 mL of a 50 µM solution of RhB with the same experimental conditions, i.e., 10 mg catalyst and 0.5 mL H_2_O_2_ and was found to be efficient (Supporting Information File 1, Figure S4).This confirms that the same as-synthesized CuO nanopetals can also be optimized for the efficient degradation of hazardous dyes other than MB.

## Conclusion

In summary, we have synthesized CuO nanoflowers and nanopetals in the absence of any surfactant and/or template using a microwave-assisted wet chemical technique. These nanostructures exhibited an increased band gap with larger surface area. The CuO nanopetals, with a specific surface area ≈40 m^2^·g^−1^, have proven to be an efficient catalyst for the degradation of water pollutant, industrial dyes, even in the absence of photon irradiation (UV/visible). A corresponding mechanism for the fast degradation observes was also proposed.

## Experimental

### Materials and instrumentation

Commercial, high-grade copper sulphate (CuSO_4_·5H_2_O, 99.95%), sodium hydroxide (NaOH), ethanol (C_2_H_5_OH), acetone (C_3_H_6_O), methylene blue (MB), hydrogen peroxide (H_2_O_2_, 30%), and rhodamine B (RhB) were obtained from Sigma–Aldrich, Merck and SD Fine. CuO nanostructures were characterized by X-ray diffraction by a PANalytical high-resolution X-ray diffractometer (PW 3040/60) operated at 40 kV and 30 mA using Cu Kα X-rays (1.54 Å), energy dispersive X-ray spectroscopy using Oxford detectors, field-emission scanning electron microscopy using a Carl Zeiss SUPRA 40 instrument, and the surface area was characterized using a Quantachrome ChemBET TPR/TPD analyzer. The optical properties were analyzed using a UV–vis absorption spectrophotometer by Schimadzu 1800.

### Material synthesis

In the present study, the microwave-assisted synthesis of CuO nanoflowers and nanopetals was carried out using a microwave-irradiated wet chemical technique. At first, equimolar solutions (0.5 M) of copper sulphate and sodium hydroxide were prepared separately in 25 mL of ethanol and were allowed to stir at room temperature for about 15 min. Thereafter, sodium hydroxide solution was added drop wise to copper sulphate solution. The resulting mixture was transferred to the microwave chamber with the reaction conditions of 700 W for 10 min or 15 min. During the reaction, the color of the solution changed initially from blue to colorless and then slowly turned black. The black colloidal solution was centrifuged to separate out the precipitates. These precipitates were then washed using double distilled water, absolute ethanol, and acetone in sequence. This procedure was repeated several times. Finally, the black powder was dried at 60 °C for 4 h and used for further characterization. The synthesis parameters such as reaction time, molar concentration of the precursors, and power of microwave irradiation were monitored so as to obtain phase-pure CuO nanoflowers and nanopetals. All the reactions were carried out using Raga’s commercial scientific microwave oven attached with a reflux system.

### Catalytic activity study

The photocatalytic and the catalytic activity under dark conditions of the as-synthesized CuO nanopetals were studied for the degradation of a common cationic dye, methylene blue (MB), in the absence and presence of H_2_O_2_, respectively. For the photocatalytic activity study, 40 mg of CuO nanopetal powder was dispersed in 40 mL of a 50 µM MB solution and allowed to stir for ≈30 min under dark conditions and was then subjected to irradiation using an incandescent lamp (Philips, 200 W) at a working distance of 100 cm. The dark catalytic study was performed with 10 mg of CuO nanopetals and varying concentrations of H_2_O_2_. During both the studies, i.e., under dark conditions and the photocatalytic study, aliquots of about 4 mL were taken out from the suspension at regular intervals and were centrifuged to filter suspended CuO powder. The MB concentration in the filtered suspension was studied with a Perkin Elmer Lambda 750 UV–vis spectrophotometer. The dark catalytic activity of the as-synthesized CuO nanopetals was also checked for the degradation of RhB under the same experimental conditions.

## Supporting Information

File 1 Additional figures.
