# Supplementary material for: Enhanced catalytic activity without the use of an external light source using microwave-synthesized CuO nanopetals
Source: Beilstein J Nanotechnol. 2017 May 30;8:1167–73. doi: 10.3762/bjnano.8.118 (PMC5480358; doi:10.3762/bjnano.8.118)
Supplement: File 1 — Additional figures. [file Beilstein_J_Nanotechnol-08-1167-s001.pdf]

Supporting Information

for

Enhanced catalytic activity without the use of an external light source using microwave-synthesized CuO nanopetals

Govinda Lakhotiya[*1,2], Sonal Bajaj[2], Arpan Kumar Nayak[1], Debabrata Pradhan[1], Pradip Tekade[2] and Abhimanyu Rana[*3]

Address: [1]Material Science Center, Indian Institute of Technology Kharagpur, Kharagpur-721302, W.B., India, [2]Jankidevi Bajaj College of Science, Wardha-442001, M.S., India and [3]MESA+ Institute for Nanotechnology, University of Twente, 7500 AE, Enschede, Netherlands

Email: Abhimanyu Rana - rana.abhimanyu@gmail.com; Govinda Lakhotiya - lakhotiya.govinda@gmail.com

*Corresponding author

Additional Figures

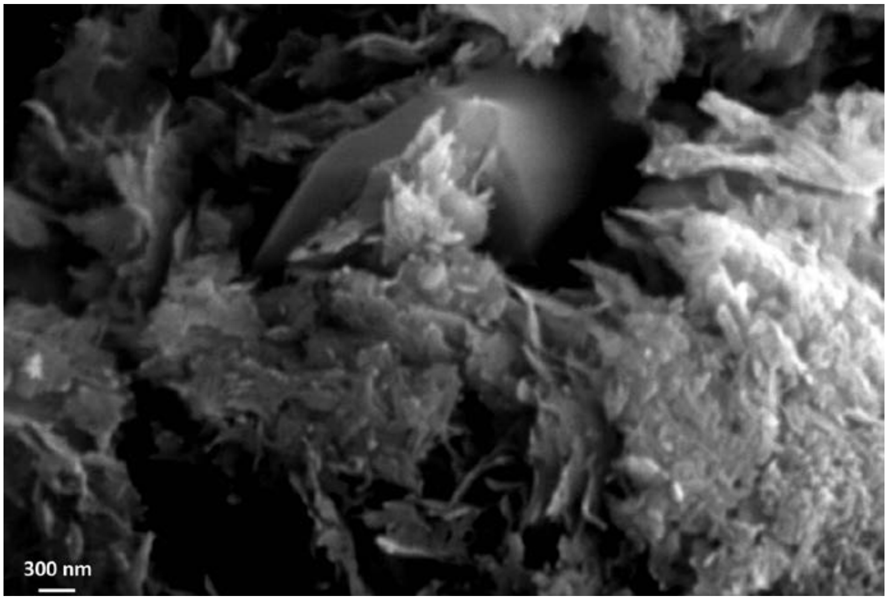

Figure S1: FESEM image of mixed phase CuO nanostructures obtained for the synthesis duration of 5 min.

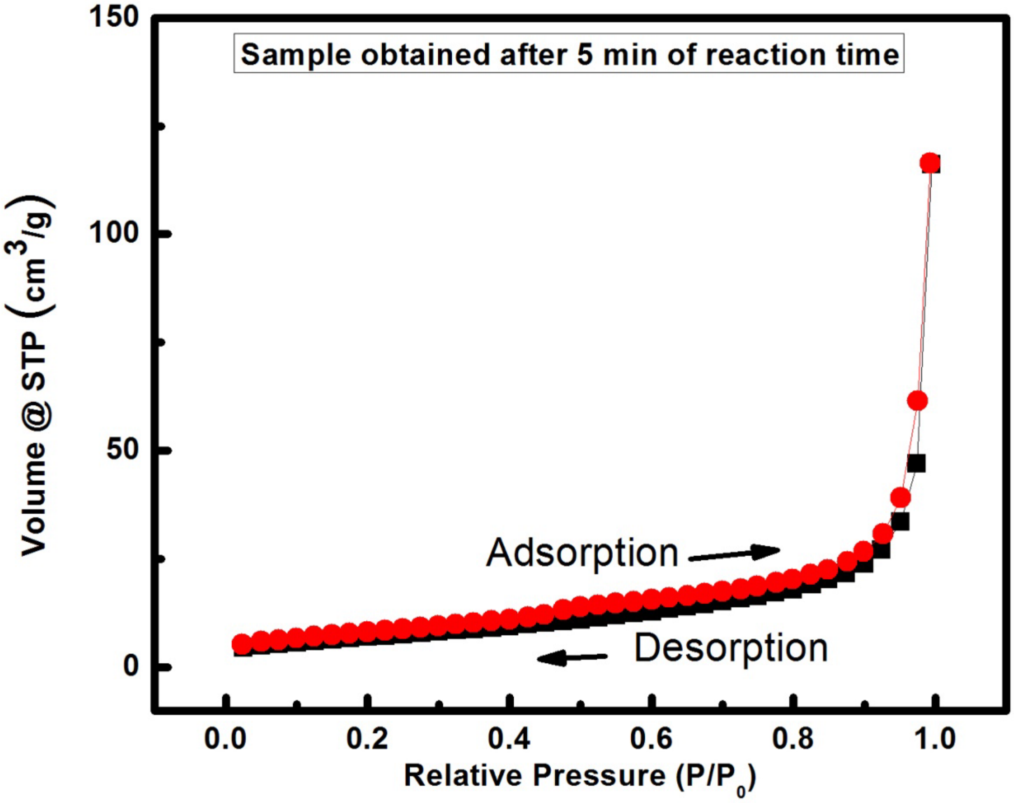

Figure S2: Nitrogen adsorption–desorption isotherm at 77 K for the sample obtained for the reaction duration of 5 min.

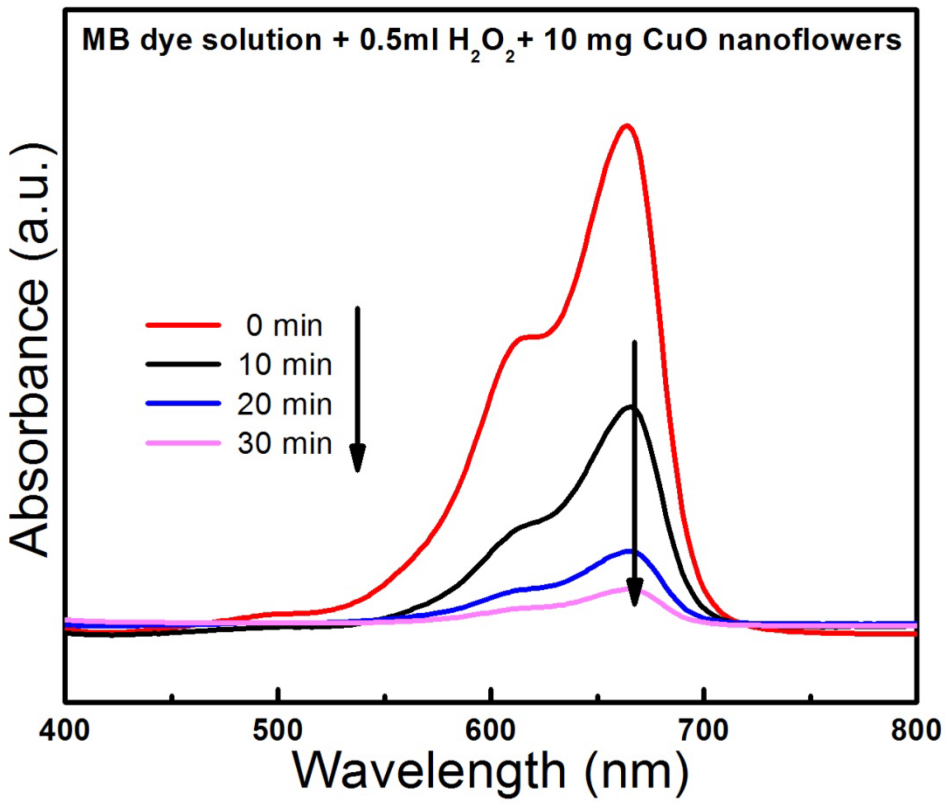

Figure S3: UV–vis absorption spectra for MB degradation for different duration for 10 mg nanoflowers of CuO and 0.5 mL H_2O_2.

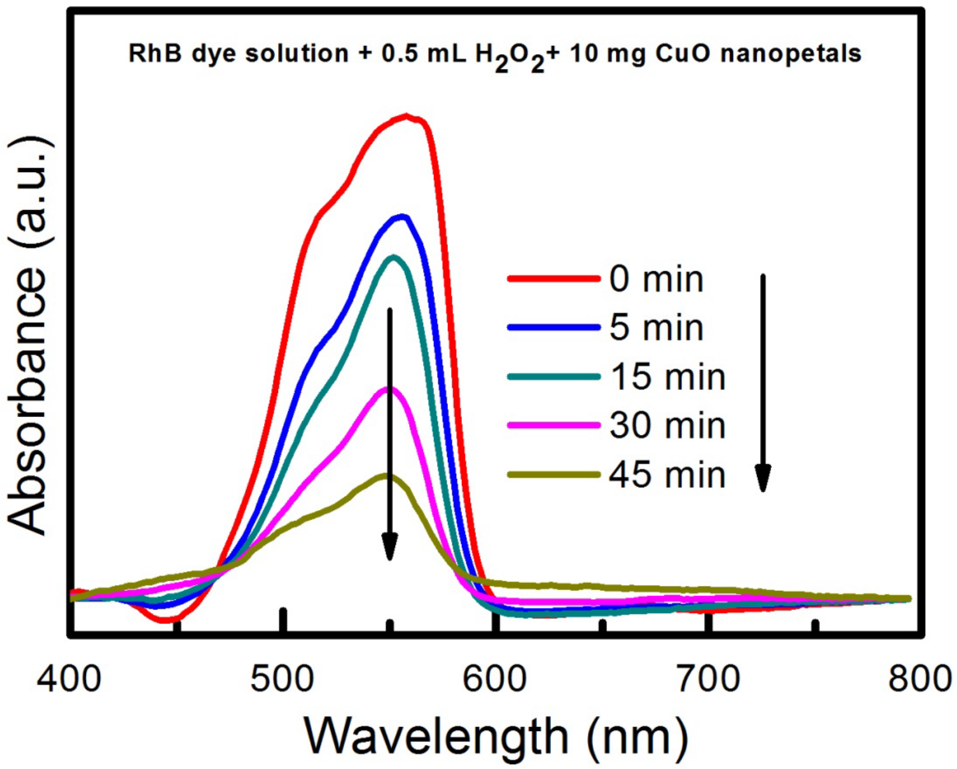

Figure S4: UV-vis absorption spectra for RhB degradation for different duration for 10 mg nanopetals of CuO and 0.5 mL H_2O_2
